# The Tolerance of Salinity in Rice Requires the Presence of a Functional Copy of *FLN2*

**DOI:** 10.3390/biom10010017

**Published:** 2019-12-20

**Authors:** Guang Chen, Jiang Hu, Liuliu Dong, Dali Zeng, Longbiao Guo, Guangheng Zhang, Li Zhu, Qian Qian

**Affiliations:** State Key Laboratory of Rice Biology, China National Rice Research Institute, Hangzhou 310006, China; hujiang588@163.com (J.H.); 17853482113@163.com (L.D.); dalizeng@126.com (D.Z.); guolongbiao@caas.cn (L.G.); zhangguangheng@126.com (G.Z.)

**Keywords:** *Oryza sativa*, FLN2, sugar partitioning, plant growth, salinity hypersensitivity

## Abstract

A panel of ethane-methyl-sulfonate-mutagenized *japonica* rice lines was grown in the presence of salinity in order to identify genes required for the expression of salinity tolerance. A highly nontolerant selection proved to harbor a mutation in *FLN2*, a gene which encodes fructokinase-like protein2. Exposure of wild-type rice to salinity up-regulated *FLN2*, while a CRISPR/Cas9-generated *FLN2* knockout line was hypersensitive to the stress. Both ribulose 1,5-bisphosphate carboxylase/oxygenase activity and the abundance of the transcript generated by a number of genes encoding components of sucrose synthesis were lower in the knockout line than in wild-type plants’ leaves, while the sucrose contents of the leaf and root were, respectively, markedly increased and decreased. That sugar partitioning to the roots was impaired in *FLN2* knockout plants was confirmed by the observation that several genes involved in carbon transport were down-regulated in both the leaf and in the leaf sheath. The levels of sucrose synthase, acid invertase, and neutral invertase activity were distinctly lower in the knockout plants’ roots than in those of wild-type plants, particularly when the plants were exposed to salinity stress. The compromised salinity tolerance exhibited by the *FLN2* knockout plants was likely a consequence of an inadequate supply of the assimilate required to support growth, a problem which was rectifiable by providing an exogenous supply of sucrose. The conclusion was that FLN2, on account of its influence over sugar metabolism, is important in the context of seedling growth and the rice plant’s response to salinity stress.

## 1. Introduction

Soil salinity represents a leading constraint over the growth and yield of most crop species [1]. Drought and salinity stress both induce tissue dehydration, but a prolonged exposure to salinity additionally imposes hyperionic and hyperosmotic stress [2]. Some plants are able to prevent the entry of toxic ions and/or to restrict their accumulation in the cytoplasm [3]; others resort to osmotic adjustment by accumulating a variety of metabolites, including sugar, in an attempt to maintain their hydration status [4]. Sugar, synthesized by plants through their photosynthetic activity and used as an energy source to support growth and development [5], can accumulate to a relatively high level in the plant’s vacuoles. In bread wheat plants exposed to either drought or salinity stress, the vacuolar content of glucose, fructose, sucrose, and fructan has been shown to be greater in salinity-tolerant varieties than in sensitive ones [6]. In *Populus euphratica* plants challenged by salinity, the soluble sugar content of young leaves increases, while that in mature leaves decreases [7]. Maize plants able to form mycorrhizal associations appear to be more tolerant of soil salinity than nonassociators, because they are able to maintain a higher tissue level of soluble sugar [8].

Plant SUTs (sucrose transporters) and SWEETs (hexose and sucrose transporters) participate not only in the loading of sucrose into the phloem, but also in the accumulation of soluble sugars in sink organs [9]. The *Arabidopsis thaliana* genome harbors 17 genes encoding a SWEET, categorized into four phylogenetic clades [10], while plant SUTs have been classified into five major clades [11]. Members of the SUT1 and SUT3 clades are responsible for phloem loading or for sucrose downloading into sink organs [12], while SUT2 members function not only as sucrose transporters but also as low-affinity sugar sensors [13]. Some members of the SUT4 clade, for example, *A. thaliana* AtSUT4, barley HvSUT2, rice SUT2, bread wheat TaSUT2, *Lotus japonicus* LjSUT4, and poplar (*Populus tremula*) PtaSUT4, localize to the vacuolar membrane and function as sucrose/H^+^ symporters [14,15,16]. According to Schulze et al. [13] and Reinders et al. [9], it is possible that HvSUT2, LjSUC4, and AtSUC4 are also responsible for the release of sucrose from the vacuole. In various plant species, *SUT* genes can be induced by a range of abiotic stress factors [17], while transgenic plants constitutively expressing *SUT* genes not only exhibit an enhanced accumulation of sucrose and soluble sugars, but are also more tolerant of abiotic stress [16,18,19]. Among the monosaccharide transporters, AtTMT1, AtTMT2, and AtTMT3 all contribute to vacuolar glucose import, with AtTMT1 playing a major role during the plants’ stress response [20]. The monosaccharide/proton symporter gene *AtSTP13* is notably induced by salinity and drought stress [21] and the rice gene *OsGMST1* is induced by salinity stress [22].

The present paper describes a successful attempt to identify and characterize genes in *japonica* type rice (cv. Nipponbare) which are important for the expression of salinity tolerance. The approach taken was to search a population of ethane-methyl-sulfonate-mutagenized plants for individuals which had become sensitive to salinity stress. Following the selection of one such individual, the identity of the mutated gene was determined and the biochemical and phenotypic consequences of the mutation were investigated.

## 2. Materials and Methods

### 2.1. Plant Materials and Growing Conditions

The mutants screened for their response to salinity stress were a subset of 500 lines out of a collection of some 50,000 generated following ethane methyl sulfonate (EMS) mutagenesis of cv. Nipponbare. The collection includes lines selected on the basis of the expression of a stably inherited variant phenotype, as well as lines which express no abnormal phenotype when the plants are grown under standard conditions, but do so when exposed to specific treatments [23]. Seedlings were grown under a 12 h photoperiod (100 µmol m^−2^ s^−1^ light intensity) at 25 °C on solidified (1% agar) half-strength Murashige and Skoog medium (1/2MS) supplemented with 3% *w*/*v* sucrose containing 100 mM NaCl (Sinopharm Chemical Reagent Co., Ltd, Shanghai, China) for 14 days, at which point each line’s germination rate, growth, and extent of chlorosis were assessed. To assess the effect on seedling growth and salinity tolerance of supplying sucrose, wild-type (WT) and CRISPR/Cas9-mediated *FLN2* knock-out mutants (KO) (see next section) were raised for 14 days under the same conditions as above on either sucrose-free 1/2MS (-S-N), 1/2MS containing 3% *w*/*v* sucrose (+S-N), sucrose-free 1/2MS containing 100 mM NaCl (-S+N), or 1/2MS containing 3% *w*/*v* sucrose and 100 mM NaCl (+S+N). At the end of the period, shoot and root dry biomass were measured and the architecture of the root system was recorded. The differential response of KO and WT adult plants to salinity stress was assessed by raising the material in normal International Rice Research Institute (IRRI)nutrient solution [24] for six weeks under a 14 h photoperiod (100 µmol m^−2^ s^−1^ light intensity), a constant temperature of 25 °C, and relative humidity of ~70%. The nutrient solution was replaced every 2 days. At the end of the six weeks, the plants were transferred into the same nutrient solution containing 150 mM NaCl where they remained for 4 days. Leaf blades, leaf sheaths, and roots were harvested at the end of the treatment and used for various physiological and genetic assays. The experiment was carried out in triplicate, with each replicate comprising five individuals per genotype per treatment.

### 2.2. Generation of KO Lines

A targeted deletion vector was constructed using the CRISPR/Cas9 system [23]. The target sequence was 5′-TCAAGTGATGATGAGAGTGA, and the vector was introduced into *Agrobacterium tumefaciens* strain EHA105 and thence into cv. Nipponbare plants, as described by Chen et al. [25]. The resulting regenerants were validated by sequencing PCR amplicons generated using the primer pair 5′-GTTACTAGATCGGGCCCAGGAATCTTTAAACATACGAACAGATCACT and 5′-AGCTTGCATGCCTGCAGGGTAAAACGGAGGAAAATTCCATC.

### 2.3. Quantitative Real-Time PCR (qRT-PCR) Assay

The procedures used to conduct qRT-PCR assays followed those given by Chen et al. [26]. RNA was extracted from leaf blades, leaf sheaths, and roots of WT and KO line plants grown either under nonstressed conditions or in the presence of NaCl. The gene *UBQ5* (*LOC_01g22490*) was chosen as the reference sequence, and relative transcript abundances were calculated following the suggestion of Chen et al. [27]. The sequences of the various primers used are listed in Table S1.

### 2.4. Analysis of Enzyme Activities and Sugar Content

WT and KO leaves and roots were snap-frozen and ground to a powder, which was stored at −80 °C. Leaf ribulose 1,5-bisphosphate carboxylase/oxygenase (Rubisco) activity was determined using a commercial kit (Comin biotechnology Co. Ltd., Suzhou, China). The sucrose content and enzyme (sucrose synthase (SS), soluble acid invertase (AI), and neutral invertase (NI)) activity in the leaf and root were determined following methods described by Chen et al. [28].

### 2.5. Phloem Export of Sucrose

Phloem exudates were collected from leaf samples using the EDTA method, as described by Chen et al. [28]. Cut ends of the leaves were dipped into 20 mL 30 mM EDTA solution (pH 7, Sinopharm Chemical Reagent Co., Ltd, Shanghai, China) and held in the dark for 15 min. To avoid contamination with xylem exudate, the initial solution was discarded, and the leaves were rinsed and transferred to a 10 mL volume of fresh solution. The leaves were kept in the dark in an air-tight chamber at ambient temperature. After 4 h, the sucrose concentration in the solution was measured using the sucrose assay kit described above.

### 2.6. Characterization of Root System Architecture

The root system architecture was characterized as described by Chen et al. [29], based on the use of WinRhizo V4.0b software (Regent Instruments Inc., Quebec, QC, Canada, http://regent.qc.ca/). Five seedlings per line were analyzed.

### 2.7. Statistical Analysis

Analyses of variance were carried out using routines implemented in SPSS v10 software (SPSS Inc., Chicago, IL, USA). Statistically significant differences either between the performance of KO line and WT plants or between different treatments were determined based on Tukey’s test.

## 3. Results

### 3.1. Selection of a Salinity-Hypersensitive Mutant

**Figure 1:**
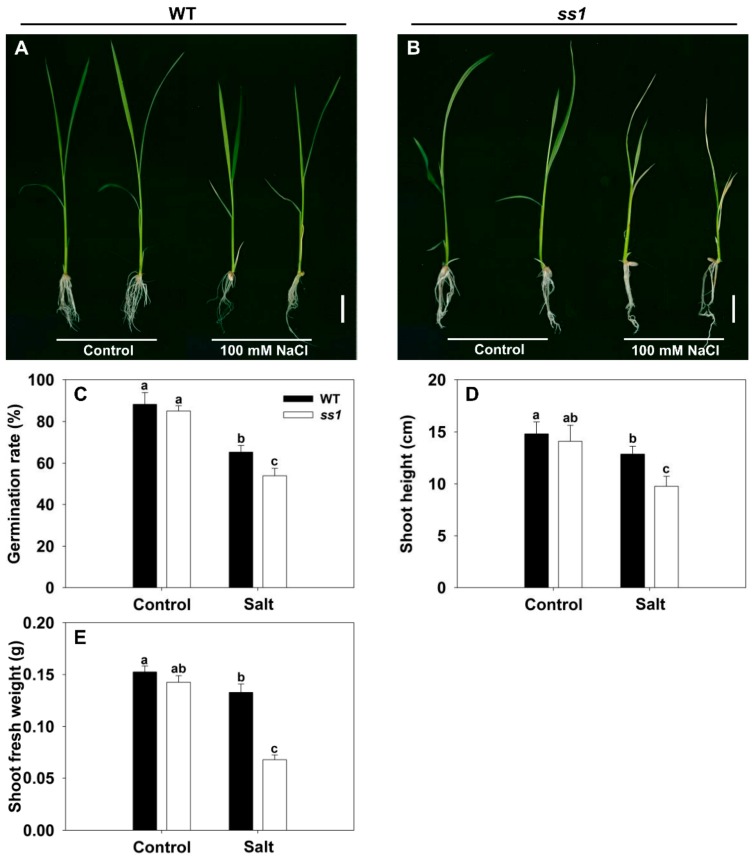
The phenotypic response of the *ss1* mutant and wild-type (WT) cv. Nipponbare to salinity stress. (**A**,**B**) The appearance of 14-day-old seedlings of (**A**) WT and (**B**) *ss1* mutant challenged with 100 mM NaCl. Bar: 2 cm. (**C**–**E**) Quantification of the response in terms of (**C**) the rate of germination, (**D**) the elongation of the shoot, and (**E**) the fresh weight of the shoot. Control: nonstressed seedling, Salt: seedlings exposed to 100 mM NaCl. Data are given in the form mean ± SE (*n* = 5). Means differing significantly (*p* < 0.05) from one another are indicated by a different lowercase letter.

The screening of mutagenized cv. Nipponbare succeeded in identifying a particularly salinity-sensitive line, which has been designated *ss1*. There was no discernible difference between the growth of WT and *ss1* plants in the absence of salinity stress, but when challenged with 100 mM NaCl, *ss1* plants—but not WT plants—wilted heavily and became chlorotic (Figure 1A,B). In the absence of NaCl stress, the rate of germination of the mutant was indistinguishable from that of WT, but in its presence, while germination was inhibited for both genotypes, WT grains germinated more freely (Figure 1C). Shoot growth was suppressed by salinity for both WT and *ss1* seedlings, but the extent of the reduction induced in both shoot length and fresh weight was greater for the *ss1* seedlings (Figure 1D,E). This mutant has previously been characterized by Qiu et al. [23] as carrying a lesion in a gene encoding fructokinase-like protein2 (FLN2), a protein not known to be involved in the response of rice to salinity stress. Here, CRISPR/Cas9 gene editing was used to generate lines in which *FLN2* was knocked out in order to investigate in detail the mechanistic basis of FLN2’s contribution to salinity stress tolerance.

### 3.2. FLN2 Is Inducible by Salinity Stress and Its Absence Leads to a Loss in Tolerance

**Figure 2:**
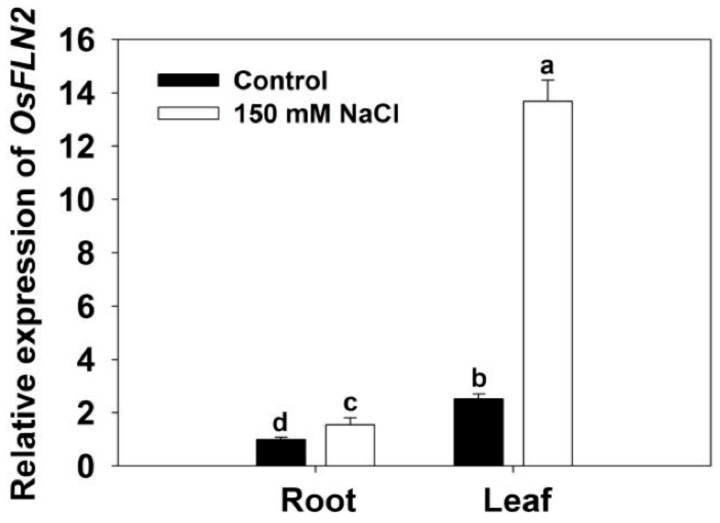
The abundance of *FLN2* transcript in the root and leaf of six-week-old cv. Nipponbare plants exposed for 4 days to either 0 or 150 mM NaCl. Data, acquired using a qRT-PCR assay, are given in the form mean ± SE (*n* = 3). Means differing significantly (*p* < 0.05) from one another are indicated by a different lowercase letter.

After the 4 days exposure of six-week-old WT plants to 150 mM NaCl, the abundance of *FLN2* transcript rose 0.6 fold in the root and 4.5 fold in the leaf (Figure 2).

**Figure 3:**
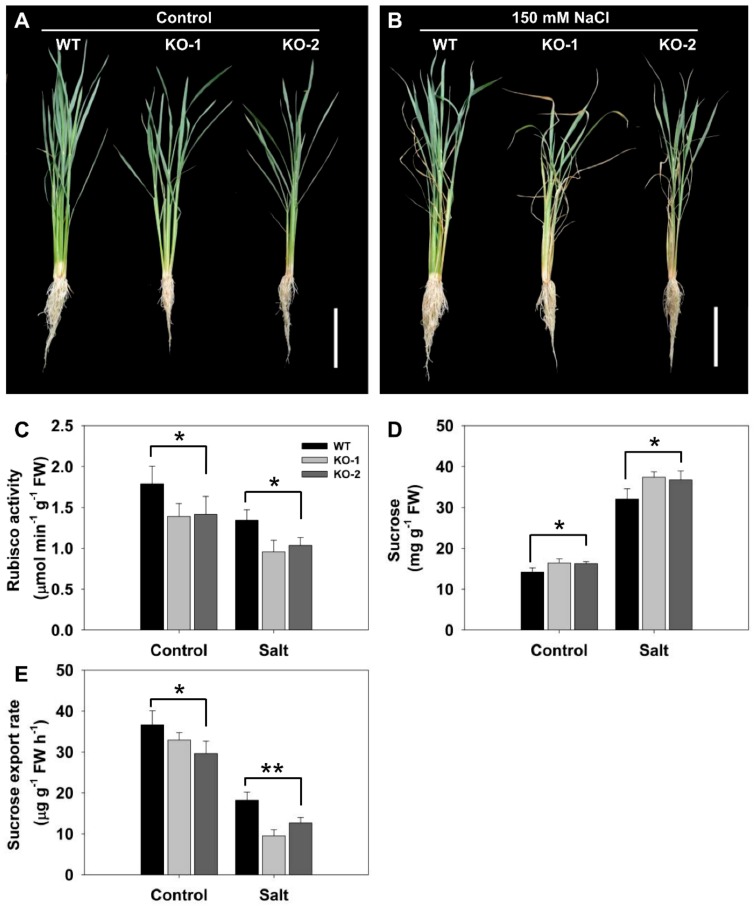
Knocking out *FLN2* increases the sensitivity of rice to salinity stress. The appearance of six-week-old plants of WT and two *FLN2* knockout lines (KO-1 and -2) exposed for 4 days to (**A**) 0 mM NaCl or (**B**) 150 mM NaCl. Bar: 10 cm. (**C**–**E**) Quantification of the response in terms of (**C**) leaf Rubisco activity, (**D**) leaf sucrose content, and (**E**) the rate of sucrose export from the phloem. Data are given in the form mean ± SE (*n* = 5). *, **: The performance of the KO line plants differed significantly (*p* < 0.05, *p* <0.01) from that of WT. FW: fresh weight.

The importance of *FLN2* expression in the plant’s ability to tolerate the stress was explored by generating CRISPR-Cas9-induced *FLN2* knockout (KO) lines. In line KO-1, a thymine was inserted within the target sequence, while in line KO-2, a guanine was inserted (Figure S1). Both events resulted in a frameshift, leading to the premature termination of the *FLN2* transcript. Exposure to salinity stress inhibited the growth of both KO line plants more strongly than was the case for WT plants (Figure 3A).

### 3.3. The Effect of Knocking Out FLN2 on Sucrose Metabolism and Partitioning

*RbcL*, which encodes the large subunit of Rubisco, is known to be transcribed at a much lower level in *ss1* than in WT plants [23]; this proved to also be the case for KO line plants grown under nonstressed conditions (Figure 3C). Exposure to salinity stress significantly reduced Rubisco activity in both WT and KO line plants, but the magnitude of the decline was greater for the latter (Figure 3C). In the absence of salinity stress, KO line plants accumulated on average 14% more sucrose in their leaves than did WT plants, rising to 17% for plants challenged with NaCl (Figure 3D). The quantity of sucrose exported through the phloem was substantially reduced by the absence of *FLN2*: the amount exported per hour per gram of leaf fresh weight of KO line plants was 10–19% lower than the level exported by WT plants in the absence of stress, rising to 30–48% in the stressed plants (Figure 3E).

**Figure 4:**
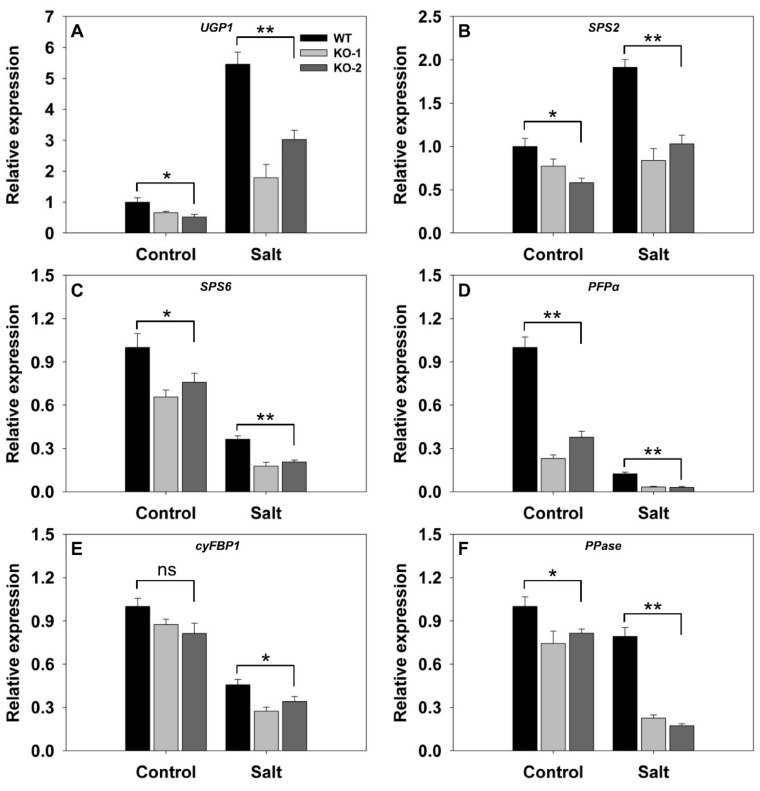
Transcriptional profiling, derived from qRT-PCR assays, of key genes involved in sucrose synthesis in the leaves of two *FLN2* knockout line (KO-1 and -2) and WT plants in response to salinity stress. Six-week-old plants were exposed for 4 days to either 0 mM NaCl (Control) or 150 mM NaCl (Salt). The genes assayed were (**A**) *UGP1*, (**B**) *SPS2*, (**C**) *SPS6*, (**D**) *PFPα*, (**E**) *cyFBP1*, and (**F**) *PPase*. Data are given in the form mean ± SE (*n* = 3). *, **: The performance of the KO line plants differed significantly (*p* < 0.05, *p* < 0.01) from that of WT; ns: means did not differ significantly.

A comparison was then made of the transcriptional activity in the leaves with respect to a number of genes encoding components of sucrose synthesis. In nonstressed plants, with the exception of *cyFBP1* (*LOC_01g0866400*) which was transcribed equally in WT and KO line plants, the genes were all more strongly transcribed in WT than in KO line plants (Figure 4). In response to salinity stress, *UGP1* (*LOC_01g0264100*) and *SPS2* (*LOC_02g0184400*) were both up-regulated more strongly in WT than in KO line plants (Figure 4A,B). Meanwhile, the stress down-regulated *SPS6* (*LOC_06g0634800*), *PFPα* (*LOC_02g0714200*), *cyFBP1*, and *PPase* (*LOC_01g0866500*) in both WT and KO line plants, but the extent of the suppression was greater in the latter (Figure 4C–F). The most strongly affected gene was *PFPα,* which was barely transcribed in the KO line plants (Figure 4D).

**Figure 5:**
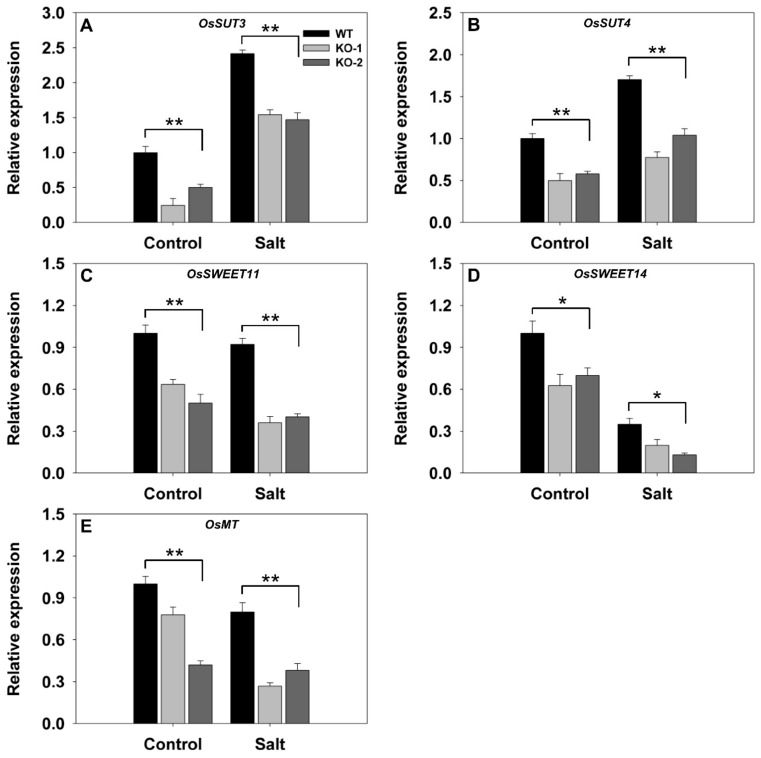
Transcriptional profiling, derived from qRT-PCR assays, of key genes involved in carbon transport in the leaves of two *FLN2* knockout line (KO-1 and -2) and WT plants in response to salinity stress. Six-week-old plants were exposed for 4 days to either 0 mM NaCl (Control) or 150 mM NaCl (Salt). The genes assayed were (**A**) *SUT3*, (**B**) *SUT4*, (**C**) *SWEET11*, (**D**) *SWEET14*, and (**E**) *MT*. Data are given in the form mean ± SE (*n* = 3). *, **: The performance of the KO line plants differed significantly (*p* < 0.05, *p* < 0.01) from that of WT.

A similar comparison for a set of five genes involved in carbon transport showed that all were transcribed less abundantly in nonstressed KO line plants than in WT plants grown under nonstressed conditions (Figure 5). The effect of the stress was to up-regulate both *SUT3* (*LOC_10g0404500*) and *SUT4* (*LOC_02g0827200*) more strongly in the WT than in the KO line plants (Figure 5A,B). At the same time, the stress down-regulated *SWEET11* (*LOC_*08g0535200), *SWEET14* (*LOC_11g0508600*), and *MT* (*LOC_04g0602400*) in both the WT and KO line plants, but the magnitude of the suppression was greater in the latter (Figure 5C–E).

**Figure 6:**
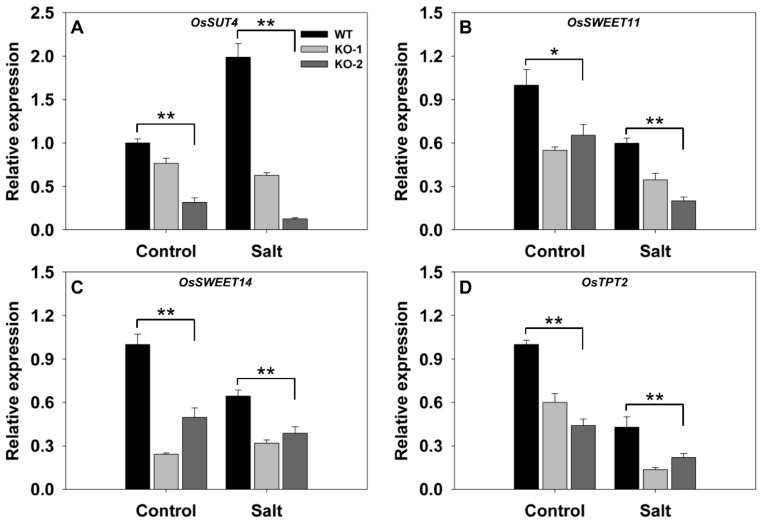
Transcriptional profiling, derived from qRT-PCR assays, of key genes involved in carbon transport in the leaf sheaths of two *FLN2* knockout line (KO-1 and -2) and WT plants in response to salinity stress. Six-week-old plants were exposed for 4 days to either 0 mM NaCl (Control) or 150 mM NaCl (Salt). The abundances of transcript generated from (**A**) *SUT4*, (**B**) *SWEET11*, (**C**) *SWEET14*, and (**D**) *TPT2* were determined. Data are given in the form mean ± SE (*n* = 3). *, **: The performance of the KO line plants differed significantly (*p* < 0.05, *p* < 0.01) from that of WT.

The transcriptional response in the leaf sheath to knocking out *FLN2* was also considered. In plants grown under nonstressed conditions, *SUT4*, *SWEET11*, *SWEET14*, and *TPT2* (*LOC_05g0241200*) were all less abundantly transcribed (although to differing extents) in the KO line plants (Figure 6). The imposition of salinity stress down-regulated *SWEET11, SWEET14*, and *TPT2* in WT leaf sheaths (Figure 6B–D) and up-regulated *SUT4* (Figure 6A). Meanwhile, in salinity-stressed KO line plants, all four of these genes were down-regulated (Figure 6): compared to WT sheaths, their relative transcript abundances were, respectively, 19%, 45%, 55%, and 41% (Figure 6).

### 3.4. The Effect of Knocking Out FLN2 on the Biochemistry of the Root

**Figure 7:**
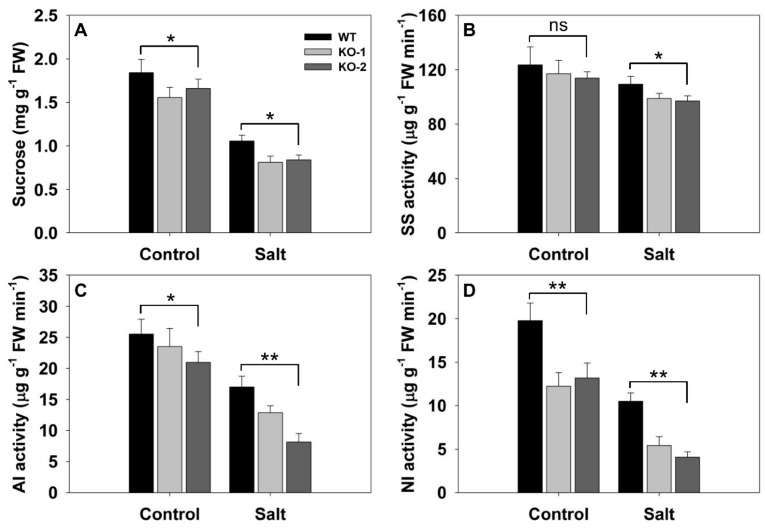
The effect of knocking out *FLN2* on root biochemistry. Six-week-old plants of WT and two *FLN2* knockout lines (KO-1 and -2) were exposed for 4 days to either 0 mM NaCl (Control) or 150 mM NaCl (Salt). (**A**) Sucrose content, (**B**–**D**) enzyme activity: (**B**) sucrose synthase (SS), (**C**) soluble acid invertase (AI), and (**D**) neutral invertase (NI). Data are given in the form mean ± SE (*n* = 5). *, **: The performance of the KO line plants differed significantly (*p* < 0.05, *p* < 0.01) from that of WT; ns: means did not differ significantly. FW: fresh weight.

In the absence of stress, the sucrose content of the roots of KO line plants was significantly lower than that of WT roots. In its presence, the sucrose content of the roots of WT plants was reduced less severely than was that of the roots of KO line plants; as a result, the sucrose content of the KO line plants’ roots was only ~78% that of the WT plants’ roots (Figure 7A). When the activity of the three enzymes central to sucrose degradation was monitored, it was apparent that, in the absence of stress, WT and KO line roots shared a comparable level of SS activity (Figure 7B), but the levels of AI and NI activity were lower in the KO line roots (Figure 7C,D). The imposition of salinity stress reduced the activity of all three enzymes in WT, but did so even more strongly in the KO line roots (Figure 7B–D).

### 3.5. The Exogenous Supply of Sucrose Mitigates the Adverse Effects Induced by the Absence of FLN2

**Figure 8:**
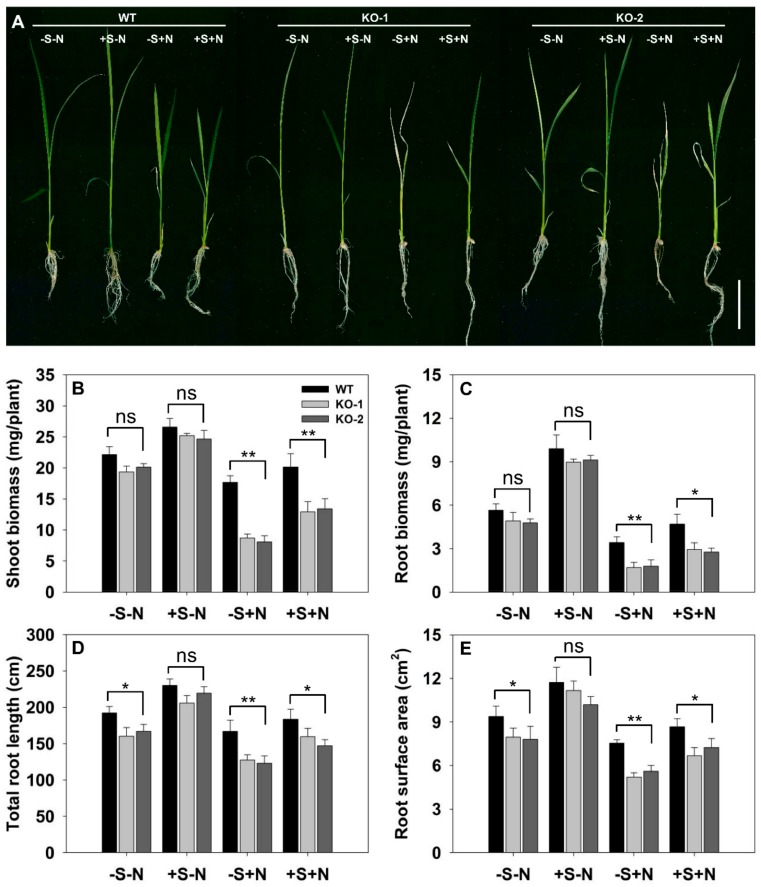
Exogenously supplying sucrose reduces the salinity sensitivity of *FLN2* knockout line plants. Seedlings were raised for 14 days on one of four contrasting media: -S-N: sucrose-free half-strength Murashige and Skoog medium (1/2MS), 0 mM NaCl; +S-N: 1/2MS, 3% w/v sucrose, 0 mM NaCl; -S+N: sucrose-free 1/2MS, 100 mM NaCl; +S+N: 1/2MS, 3% w/v sucrose, 100 mM NaCl. (**A**) Appearance of the seedlings. Bar: 2 cm. (**B**–**E**) Quantification of (**B**) shoot biomass, (**C**) root biomass, (**D**) root length, and (**E**) root surface area. Data are given in the form mean ± SE (*n* = 5). *, **: The performance of the KO line plants differed significantly (*p* < 0.05, *p* < 0.01) from that of WT; ns: means did not differ significantly.

The association between the action of FLN2, sugar metabolism, plant growth, and salinity tolerance was explored by supplying plants with sucrose. The accumulation of dry weight by both the root and the shoot of KO line plants and WT was comparable for seedlings raised on the -S-N medium. The growth of both the root and the shoot was significantly greater than this on the medium which provided a source of sucrose (+S-N), but there was no significant difference between the performance of the KO line and the WT plants grown on this medium (Figure 8A–C). The appearance of the root system, however, did differ between WT and KO line seedlings grown on the -S-N medium (Figure 8D,E). Supplying sucrose clearly lifted the inhibition of root growth experienced by the KO line roots, since for plants grown in the +S-N medium, there was no genotypic difference with respect to either the total root length or the root surface area (Figure 8D,E). The imposition of salinity stress had a pronounced negative effect on the growth of both WT and KO line seedlings, but the effect was less severe for the former (Figure 8). When grown without sucrose supplementation but in the presence of NaCl, the KO line seedlings’ shoot biomass, root biomass, total root length, and root surface area were, respectively, 48%, 51%, 75%, and 72% of those achieved by WT seedlings (Figure 8B–E); when sucrose was provided exogenously, these differences were reduced to, respectively, 35%, 40%, 16%, and 20%.

## 4. Discussion

Collections of rice mutants have been widely used to search for genes influencing the abiotic stress response [23,27,30]. Here, an EMS mutant collection library was successfully used to identify the salinity-stress-hypersensitive line *ss1*. This mutant has been shown to carry a lesion in *FLN2* and to respond to high temperature (32 °C) conditions by forming albino seedlings [23]. While there was no phenotypic differentiation between *ss1* and WT plants raised at 25 °C in the absence of salinity stress (1/2MS supplemented with 3% w/v sucrose) (Figure 1A,B), the response of the mutant to salinity was quite distinct from that of the WT, showing that its sensitivity to salinity cannot be associated with the albino trait.

An important observation was that the level of Rubisco activity in the leaves of the *FLN2* knockout line plants was significantly lower than that in WT leaves (Figure 3C), consistent with the previous finding that the *ss1* mutant’s leaves generate both less *RbcL* transcript and less RbcL protein than do WT leaves [23]. At the same time, the intensity of transcription of key genes involved in sucrose synthesis was significantly reduced in the mutants’ leaves (Figure 4). A decline in Rubisco activity and sucrose synthesis would be expected to restrict the accumulation of sucrose in the leaf, but the experimental outcome was just the opposite, since the content of sucrose in the KO line plants’ leaves was significantly higher than that in the WT plants’ leaves (Figure 3D). This unexpected result likely reflects a lowered capacity of the KO genotypes to export sucrose through the phloem. The evidential support for this idea rests on three observations: firstly, that the measured rate of sucrose exported from the leaf (on a per unit fresh weight of leaf basis) was lower for the KO line plants’ leaves than for the WT ones (Figure 3E); secondly, that a number of genes involved in carbon transport were down-regulated in both the leaf and the leaf sheath of the KO line plants (Figure 5 and Figure 6); and lastly, that the sucrose content of the KO line plants’ root was lower than that of WT roots (Figure 7A). Restricted export of sucrose is thought to disturb the carbohydrate metabolism occurring in assimilate sink organs [28,31], as was clearly the case for sucrose hydrolysis, given that the activity of SS, AI, and NI was markedly lower in the KO line plants’ roots than in WT roots (Figure 7B–D).

The hypothesis arising from the present experiments is that the heightened sensitivity to salinity stress induced by a lack of FLN2 activity results from a disruption in the plant’s sugar metabolism. The differences between KO line and WT plants with respect to their ability to fix carbon (Figure 3C) and synthesize, export, partition, and utilize sucrose (Figure 3E and Figure 4, Figure 5, Figure 6 and Figure 7) were more pronounced in plants subjected to salinity stress than in nonstressed plants, while the KO line plants appeared to be particularly sensitive to the stress (Figure 3A and Figure 8A). Sucrose (and other sugars) are used by stressed plants as an osmoticum [16,19]: for example, plants of the halophytic species *Thellungiella halophila* respond to high levels of salinity by accumulating a significant concentration of sucrose [32]. The build-up of sugar prompted by salinity (as well as by some other abiotic stressors) is driven by the up-regulation of genes encoding components of sugar synthesis and transport [33]. The abiotic stress response of *A. thaliana* and of certain other plant species includes alterations in sucrose transport [17]. Both *AtSUC1* and *AtSUC2* are up-regulated when the plants are exposed to low temperature [34]. In *A. thaliana*, the loss-of-function of SUC4 results in an abnormal distribution of sucrose in both the shoot and the root, leading to a phenotype which is highly sensitive to abiotic stress [35]. Meanwhile Jia et al. [36] have shown that AtSUC9 mediates sucrose distribution in a way which supports the plant’s tolerance of abiotic stress resistance. Suppression of the aspen (*P. tremula*) gene *PtaSUT4* alters the plant’s responsiveness to moisture stress [15], while the over-expression in apple (*Malus × domestica*) of *MdSUT2.2* promotes tolerance to both salinity and drought [16,19]. In rice, both SUT1 and SUT2 contribute to the salinity and drought response [37,38]. Here, the transcription of *SUT3* in the leaf and *SUT4* in both the leaf and the leaf sheath was markedly reduced in plants lacking a functional copy of *FLN2*, especially in plants subjected to salinity stress (Figure 5A,B and Figure 6A); the proposition is that FLN2 protects the plant from salinity-induced damage, in part, at least, by regulating SUT-mediated sugar partitioning. Other sugar transporters, notably the SWEETs, are known to be important regulators of sugar accumulation in stressed plants [19]. The over-expression of *AtSWEET16* enhances freezing tolerance, likely through its positive effect on the accumulation of sugars in the vacuole [39]. Meanwhile, the heterologous expression in *A. thaliana* of either of the *Dianthus spiculifolius* genes *DsSWEET12* or *DsSWEET17* affects not just the plant’s sugar metabolism but also its tolerance of multiple stressors [40,41]. Here, similarly, the abundance of *SWEET11* and *SWEET14* transcript in the leaf and leaf sheath of the stressed KO line plants proved to be considerably lower than that in the tissues of stressed WT plants (Figure 5C,D and Figure 6B,C). Overall, the incapacitation of *FLN2* inhibited the movement of sugar from the source (leaf) to the sink (root) of plants exposed to salinity stress by down-regulating a number of genes encoding SUTs and SWEETs; this may well be the primary basis for the greater sensitivity to salinity shown by the KO line plants.

While sugar can be used as an osmoticum, its main role is to provide the plant with the energy it needs for growth and development [42]. One of the major determinants of salinity tolerance in rice is thought to be the plant’s inherent growth rate, since this has a large impact on how much toxic Na^+^ reaches the shoot meristem [43]. Thus, the ability to maintain rapid growth when challenged with salinity has been suggested to be a robust indicator of tolerance [44,45,46]. With respect to shoot growth in the face of salinity stress, KO line plants clearly under-performed compared with WT ones, both at the seedling (Figure 8A,B) and at the tillering (Figure 3B) stage. The root system developed by salinity-challenged KO line plants was less vigorous than that by WT plants, with respect to its dry matter accumulated, its length, and its surface area (Figure 3B and Figure 8A,C–E). This loss in vigor likely resulted from the roots receiving an inadequate supply of assimilate, given that there was a clear difference between the sucrose contents of the KO line and WT plants’ roots (Figure 7A), yet there was no such genotypic difference in root growth between seedlings raised on a salinity-free medium when sucrose was supplied exogenously (Figure 8C–E). The data are consistent with the idea that the loss of FLN2 function inhibited the growth of both the shoot and root as a result of a reduction in the supply of assimilate, a problem which was exacerbated when the plants were stressed with salinity.

## 5. Conclusions

**Figure 9:**
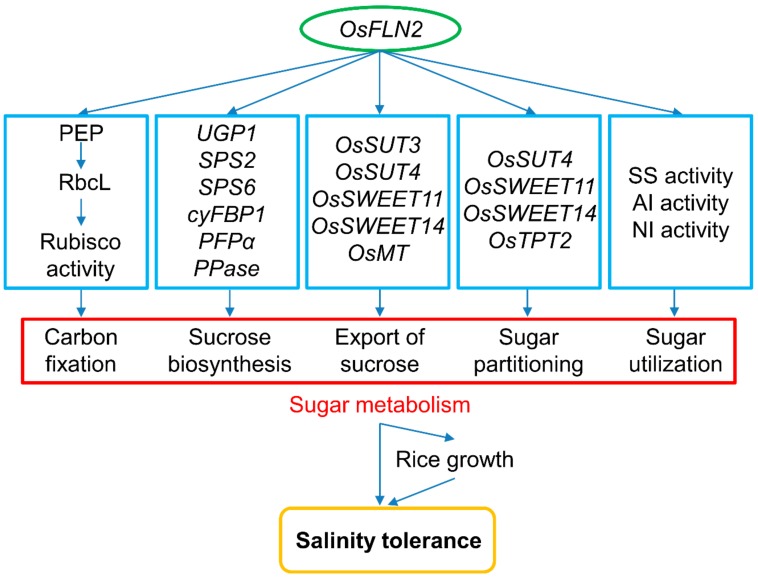
A proposed model of the interaction between FLN2, sugar metabolism, and salinity tolerance. The inactivation of *FLN2* results in a reduced level of Rubisco activity and sucrose export rate and down-regulates genes encoding enzymes involved in sucrose synthesis and transporters involved in carbon transport in the leaf, as well as several encoding sucrose transporters and carbon metabolism-related plastidic translocators in the transport phloem network. Due to the impairment of SS, AI, and NI activity in the root, sugar utilization is likely compromised, so the carbohydrate requirement for growth is not met, resulting in both growth retardation and a heightened sensitivity to salinity stress.

FLN2 represents an important component of rice’s response to salinity stress. The loss-of-function of *FLN2* could depress sugar metabolism in at least three ways: firstly, by inhibiting the activity of Rubisco and down-regulating genes encoding the components of sucrose synthesis in the leaf; secondly, by down-regulating genes encoding the components of carbon transport in the leaf and leaf sheath; and thirdly, by inhibiting the activity of SS, AI, and NI in the root (Figure 9). Given that the sink and source organs of *ss1* (and also *FLN2* knockout line) plants, especially in the presence of salinity stress, are imbalanced in terms of their sucrose content, the indication is that functional FLN2 protein protects the plant from salinity-induced damage by supporting the plant’s sugar metabolism.

## Supplementary Materials

The following are available online at https://www.mdpi.com/2218-273X/10/1/17/s1, Figure S1: Sequence confirmation of the *FLN2* mutations induced by Cas9/sgRNA, Table S1: The sequences of primers used for qRT-PCR assays.
