# Supplementary material for: The Tolerance of Salinity in Rice Requires the Presence of a Functional Copy of FLN2
Source: Biomolecules. 2019 Dec 20;10(1):17. doi: 10.3390/biom10010017 (PMC7022601; doi:10.3390/biom10010017)
Supplement: Supplementary file 1 [file biomolecules-10-00017-s001.pdf]

Supplementary Information

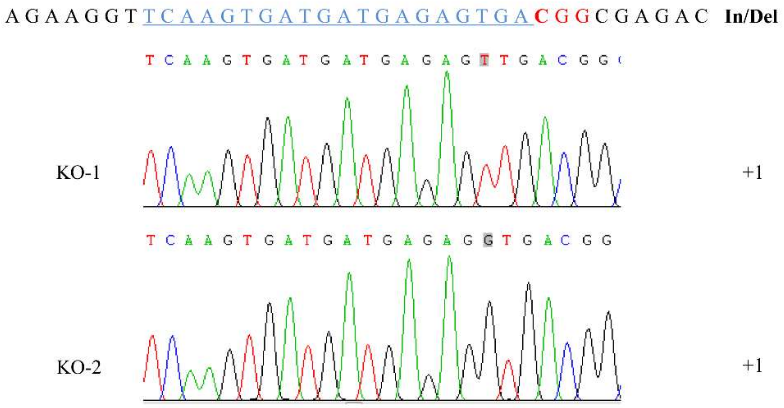

Fig. S1. Sequence confirmation of the *FLN2* mutations induced by Cas9/sgRNA. The 20 nt Cas9/sgRNA target sequence is shown underlined in blue, the PAM site is marked in red, and the inserted nucleotides in gray. The net length of the indel is shown on the right.

Table S1. The sequence of primers used for qRT-PCR assays.

Gene	Primer ID	Primer sequences
UBQ5	F(5’-3’)	CTCGCCGACTACAACATCCA
	R(5’-3’)	TCTTGGGCTTGGTGTACGTCTT
OsFLN2	F(5’-3’)	CCGAATGGCTTCTCTTCTTCTC
	R(5’-3’)	GGCTCCTGATTGAGTTGGTACTACA
UGP1	F(5’-3’)	ATGTCGTCGGAGGCGCGGG
	R(5’-3’)	CGGCGGCGGAGGAGGAGCG
SPS2	F(5’-3’)	ATGTGGTTGAACTTGCTAAA
	R(5’-3’)	GATATGGCCAAGTGCACC
SPS6	F(5’-3’)	TCTTCAGCGGCCTCCTCCGC
	R(5’-3’)	GCGCCGACTCCGACGAC
PFPα	F(5’-3’)	GTGGAAGTGATGGTCTTCTC
	R(5’-3’)	CAATGATTACTAAAGCATCC
cyFBP1	F(5’-3’)	ATGGATCACGAGGCGGACG
	R(5’-3’)	TTGTTGACGGCGGAGGCGAC
PPase	F(5’-3’)	GAGCGAGGCGGACGGAGGCG
	R(5’-3’)	GTGTCGAGGTCGTGCCAGG
OsSUT3	F(5’-3’)	CCGTCGACATGGAGCTCGA
	R(5’-3’)	AACGTACGGGGTGAGGAGA
OsSUT4	F(5’-3’)	CGCCGGCGGTGGCGGCCTCA
	R(5’-3’)	CGTGAGGAGCGAGAGCTGA
OsSWEET11	F(5’-3’)	GACGTTCTTGCAGGTGTACA
	R(5’-3’)	TAGCGGACGATGTAGGCGGC
OsSWEET14	F(5’-3’)	TTCCCAACGTGCTGGGCTTCT
	R(5’-3’)	GCACCTCGCGGGTCTTGACG
OsMT	F(5’-3’)	GCTGCCAGGCAGGAAGCT
	R(5’-3’)	GGTTCCAGTTTCACCACGACA
OsTPT2	F(5’-3’)	TAGTTGGGTAGCTGCTTTGATCGA
	R(5’-3’)	AAATGGGATGATGGAGGCTTTG
